# GAP-Seq: a method for identification of DNA palindromes

**DOI:** 10.1186/1471-2164-15-394

**Published:** 2014-05-22

**Authors:** Hui Yang, Natalia Volfovsky, Alison Rattray, Xiongfong Chen, Hisashi Tanaka, Jeffrey Strathern

**Affiliations:** Gene Regulation and Chromosome Biology Laboratory, Frederick National Laboratory for Cancer Research, Cancer Research and Development Center, Frederick, MD 21702 USA; ABCC/ ISP, SAIC-Frederick, Inc., Frederick National Laboratory for Cancer Research, Frederick, MD 21702 USA; Department of Molecular Genetics, Cleveland Clinic Lerner Research Institute, Cleveland, Ohio 44195 USA

**Keywords:** Palindrome, Gene amplification, Inversion-PCR, GAP-Seq, GAPF, Breakpoint, MCF7, Genome instability, Cancer, Human diseases

## Abstract

**Background:**

Closely spaced long inverted repeats, also known as DNA palindromes, can undergo intrastrand annealing to form DNA hairpins. The ability to form these hairpins results in genome instability, difficulties in maintaining clones in *Escherichia coli* and major problems for most DNA sequencing approaches. Because of their role in genomic instability and gene amplification in some human cancers, it is important to develop systematic approaches to detect and characterize DNA palindromes.

**Results:**

We developed a new protocol to identify palindromes that couples the S1 nuclease treated Cot0 DNA (GAPF) with high-throughput sequencing (GAP-Seq). Unlike earlier protocols, it does not involve restriction enzymatic digestion prior to DNA snap-back thereby preserving longer DNA sequences. It also indicates the location of the novel junction, which can then be recovered. Using MCF-7 breast cancer cell line as the proof-of-principle analysis, we have identified 35 palindrome candidates and physically characterized the top 5 candidates and their junctions. Because this protocol eliminates many of the false positives that plague earlier techniques, we have improved palindrome identification.

**Conclusions:**

The GAP-Seq approach underscores the importance of developing new tools for identifying and characterizing palindromes, and provides a new strategy to systematically assess palindromes in genomes. It will be useful for studying human cancers and other diseases associated with palindromes.

**Electronic supplementary material:**

The online version of this article (doi:10.1186/1471-2164-15-394) contains supplementary material, which is available to authorized users.

## Background

Long DNA palindromes are difficult to directly analyze using standard molecular genetics methods. This is because perfect and near perfect palindromes, where a sequence is immediately followed by its exact inverse complement with very little or no spacer, are able to intrastrand anneal to form hairpin structures. Palindromes longer than 200 bp cannot be amplified by traditional PCR using DNA polymerases with low strand displacement activity, nor can they be stably maintained in *Escherichia coli*. Palindromes are also underrepresented in high-throughput sequencing results generated from libraries constructed by PCR amplification or sequencing steps that involve emulsion PCR amplification (Yang H. et al., unpublished observations).

The propensity of palindromes to adopt secondary structure interferes with DNA replication, transcription and repair, and leads to genome instability [1–5]. Natural AT-rich palindromes (PATRRs) exist at sites of some recurrent chromosomal rearrangements in humans and cause genetic disorders [6–8]. Long inverted repeats that may reflect *de novo* palindromes have been found in tumor cells and cancer cell lines, and are likely drivers of gene amplification [5, 9–12]. Previous studies demonstrated that the novel junctions of palindromes contained sequences important for understanding the mechanisms that can lead to *de novo* palindrome formation [13, 14]. Due to a lack of systematic approaches to identify and characterize palindromes from genomes, little is known about the distribution of DNA palindromes nor their association with human diseases.

Genome-wide Analysis of Palindrome Formation (GAPF) is a microarray-based technique that has been used for detection of palindromic genome rearrangements in human cancers [9, 12]. It has limitations to eliminate false positive signals and it cannot predict the orientation of palindromes, making the novel junctions difficult to find. We have explored alternative methods for systematically analyzing palindromes in the genome and here we report our analysis of *de novo* DNA palindromes from the MCF-7 breast cancer cell line [15].

**Figure 1:**
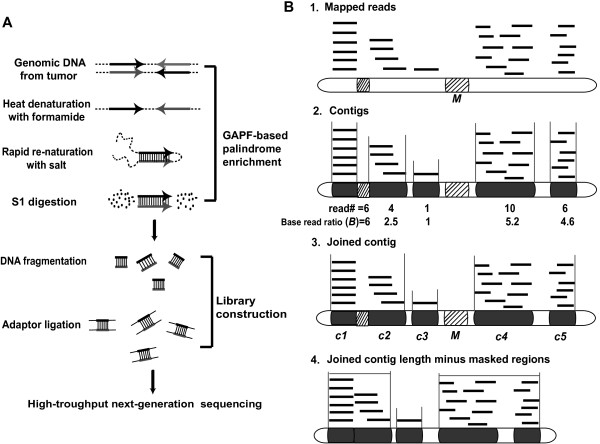
**High-throughput next generation sequencing of fast annealing DNA treated by S1 nuclease. (A)** Sequencing library construction. We prepared samples for sequencing based on efficient intrastrand base pairing of palindromes. Briefly, the genomic DNA was denatured and rapidly reannealed. After single-strand specific S1 nuclease digestion, DNA was extracted by phenol-chloroform and libraries were prepared for 454 and Illumina sequencing. **(B)** Data analysis. 1: Mapping of unique reads to human reference genome (NCBI36/hg18). Black lines represent mapped unique reads. Hatched rectangles represent regions masked by RepeatMasker (*M*). 2: Assembly of uniquely mapped reads to identify contiguous regions (contigs, black rectangles), and calculation of base read ratio (*B*) of each contig. For a contig region “*c*” with *n* mapped reads,  *.* 3: Clustering two or more contigs with a base read ratio >1.5 that are within 7.5 kb of each other to make a joined contig. 4: Determining Rank “*R*” that is the sum of all read lengths in the joined contig divided by the length of the joined contig minus the masked regions .

Chromosome rearrangements of the MCF-7 cell line have been studied by spectral karyotyping [16, 17], comparative genomic hybridization (CGH) [16, 17], array CGH [18–20], single nucleotide polymorphism (SNP) arrays [21] and gene expression arrays [18]. A BAC library from MCF-7 was generated and fully sequenced [22–24], and chromosomal breakpoints were established by high-throughput paired end-sequence profiling [25]. However, these studies would not detect palindromes due to the instability of palindromes in BAC clones and the inability of the methods used to directly sequence palindromes. The DNA Paired-End-Tag sequencing (DNA-PET) technique can detect genomic rearrangements including inverted repeats, but it cannot identify the palindrome junction or the spacer [26, 27]. Our method builds on the GAPF technique that enriches for DNA palindromes by a snap-back process of DNA denaturation and rapid reannealing (Cot0 DNA reassociation kinetics) followed by S1 nuclease digestion (an endonuclease specific for single-stranded DNA) (Figure 1A), which was first reported by Tanaka et al. using hybridization intensity to microarrays to identify candidates [9, 28, 29]. Here, we were able to significantly improve the detection of true palindromes by coupling GAPF with high-throughput sequencing, named GAP-Seq.

We used high molecular weight genomic DNA rather than enzyme digested DNA prior to DNA snap-back, because the enzyme digestion can eliminate palindromes containing the restriction site in the spacer or close to the center and can also limit the length of the signal recovered. In the analysis of our GAP-Seq data, we were able to identify true palindrome candidates by a signature pattern of read density distribution. This signature also predicted the location of the novel palindrome junction allowing junction recovery. In this study we identified 35 palindrome candidates from MCF-7 and selected the top 5 candidates for further mapping. Using inversion-PCR, we recovered 7 novel junctions that had not been identified in any previous studies despite extensive analysis. The combination of novel GAP-Seq, bioinformatics analysis and inversion PCR strategies provide a systematic approach for palindrome detection and novel junction recovery, allowing a more accurate assessment of the palindrome content in the genome.

## Results

### Bioinformatics for identification of DNA palindrome candidates

Using Roche 454 sequencing of Cot0 DNA derived from MCF-7 and IMR-90 (a normal fibroblast cell line used as a control), we obtained approximately 1 million reads (Table 1) from each cell line. The average read length for IMR-90 was 318 bp and for MCF-7 was 279 bp, and our coverage was equivalent to ~10% of the total human genome. 28% of the reads from MCF-7 sample mapped to unique regions of the human genome (Table 1). We expect DNA palindromes to be enriched among double strand sequences found in Cot0 DNA and preserved in the GAP-Seq protocol. 48% of the reads from MCF7 represented repetitive DNAs that were masked by Repeatmasker [30], 12% of the reads from MCF-7 mapped to mitochondrial DNA. The reference mitochondria DNA (mtDNA) is calculated to be about 1.6% of the human genomic DNA content (NCBI Build 36/hg18) based on average 3000 copies [31, 32]. Therefore, mtDNA in MCF-7 and IMR-90 sequencing was enriched 8–16 fold. We expected enrichment of mtDNA because covalently closed circular single strand DNAs are interlocked and stay together during denaturation.

**Table 1:** **Summary of Roche 454 data**

	^a^Reference genome (hg18) (%)	MCF7 (%)	IMR90 (%)
Mapped to unique locus	40	28	22
^b^Repeat masked	46	48	43
SINEs	13	13	9
LINEs	20	18	15
LTRs	0.02	0.03	0.02
Simple repeats	0.85	0.91	0.98
Low complexity	0.55	0.37	0.34
Satellites	3	8	10.6
Other	8.58	4.7	7.1
Mitochondrial DNA	1.6	12	25
Low copy repeats	5.2	11	7
Human but not mapped	7.2	0.6	0.6
Non-human DNA		1	2
Total # reads		934,174	1,136,611

^a^The reference genome statistics were adapted from UCSC Genome Browser (NCBI Build 36.1, Mar. 2006 Assembly, hg18). Low copy repeats (segmental duplications) were adapted from [31, 33]. We used an average of 3000 copies of mtDNA in calculating the ratio in the human genome [32].

^b^Analysis of repeat masked elements are based on RepeatMasker.

**Figure 2:**
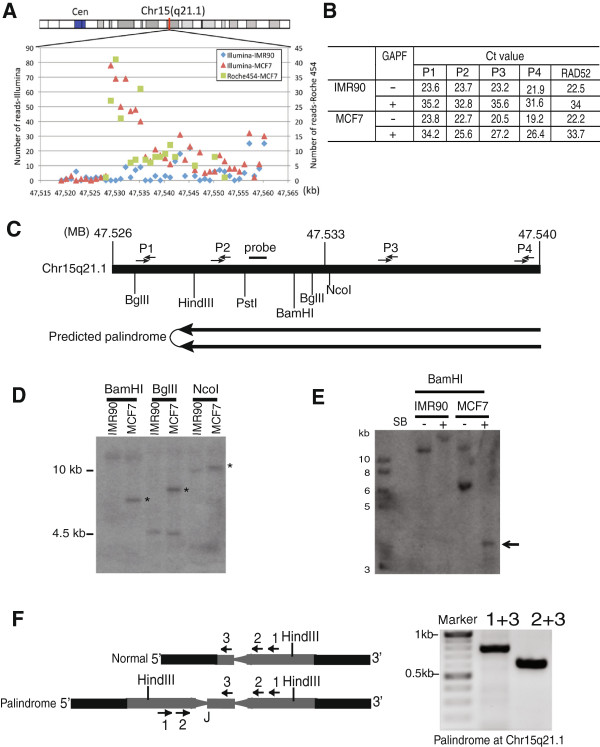
**Palindrome mapping strategy. (A)** Read density distribution in Chr15q21.1: 47,529,204-47,550,373 region shown as 1 kb bins. **(B)** qPCR analysis to monitor for palindrome enrichment and determine the directionality of the Chr15q21.1 palindrome. We calculated the amount of depletion of a specific TaqMan primer set region based on Ct value before and after GAPF protocol in both IMR-90 and MCF-7 samples. The fold enrichment is based on comparing the fold depletion among different primer sets (P1, P2, P3 and P4) relative to a single copy sequence in the genome (*RAD52*). The location of TaqMan primer sets P1, P2, P3 and P4 is indicated in Figure 2C. **(C)** Map of genomic region Chr15: 47,520,000-47,550,000 with restriction sites and primer locations. **(D)** Southern blot analysis. Genomic DNA IMR-90 and MCF-7 cells was digested with BamHI, BglII or NcoI. Asterisks (*) mark the rearranged bands from MCF-7 genomic DNA. **(E)** Snap-back (SB) southern blots of BamHI digested IMR-90 and MCF-7 DNA. Arrowhead indicates the half sized fragments after snap-back in MCF-7. **(F)** Inversion-PCR. Three primers all from the same strand in normal genomic DNA were used for PCR (Primers 1–3). Since primers 1 and 2 are located in the palindromic region, they can also be used as reverse primers. Because primer 3 is in the spacer, it is able to produce a PCR product with primer 1 or 3 containing the novel junction “J” as indicated in the figure.

**Figure 3:**
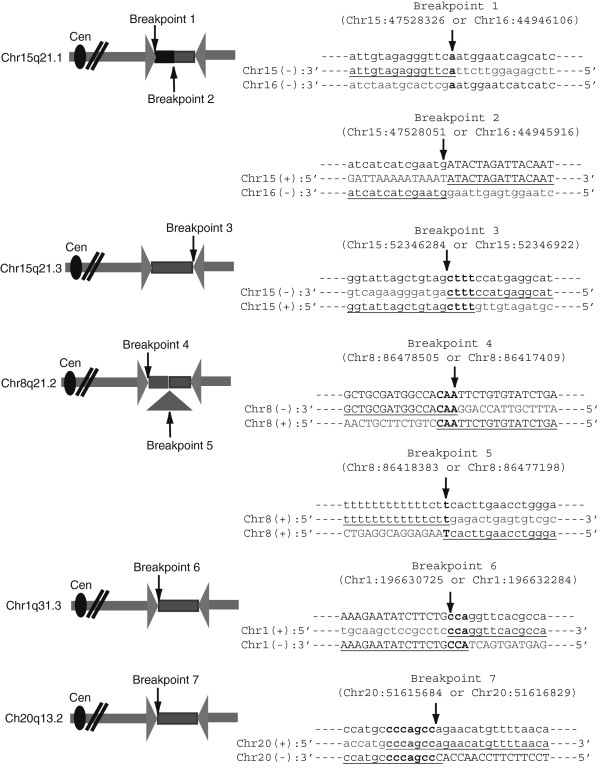
**Schematics and sequence of palindrome junctions.** Sequence analysis of the palindromic junctions identified 7 novel junctions. In each of the aligned breakpoint sequences, lowercase letters are Repeat-masker masked sequences. Uppercase letters represent unique sequences. Microhomology at the breakpoints is shown as bold letters. Insertion of Chr16 fragment at Chr15q21.1 spacer is shown as a black rectangle and deletion at Chr8q21.2 spacer is shown as a triangle in the schematics.

**Figure 4:**
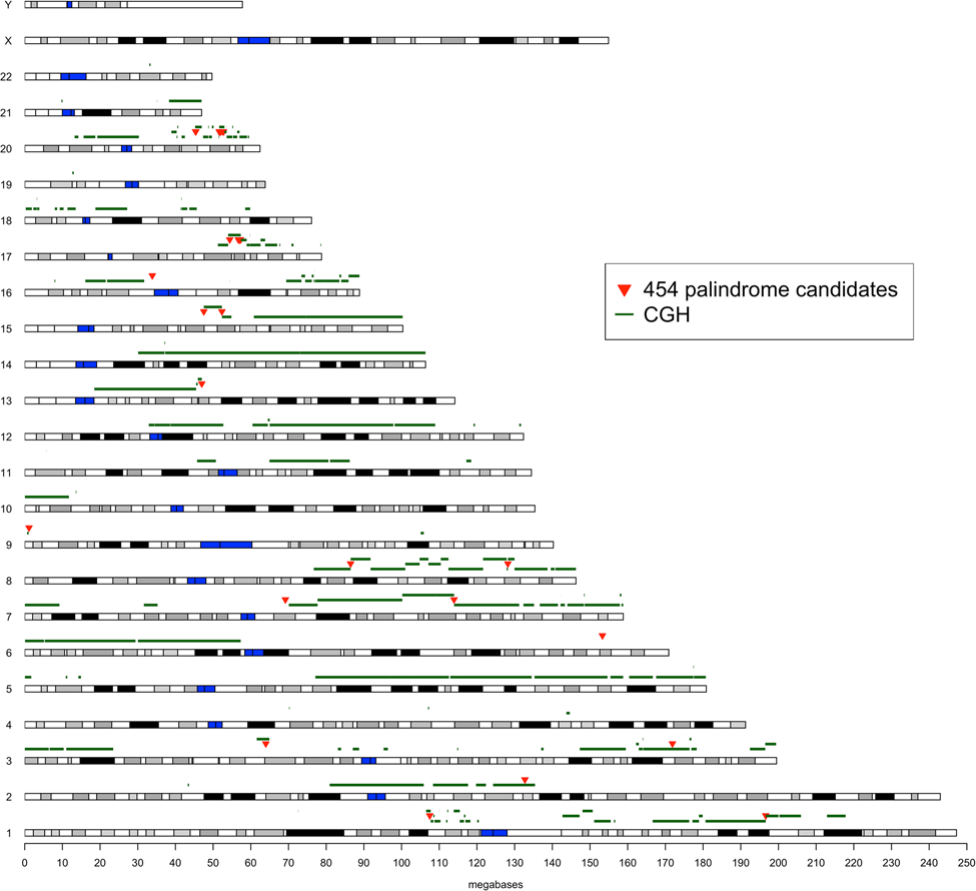
**Summary of Affymetrix SNP6 copy number gains and palindrome candidates from Roche 454 sequencing of MCF-7.** Key: red triangle-palindrome candidates from Roche 454 sequencing; green line-copy number gains based on Affymetrix SNP6 analysis; blue rectangle-centromere region. The data used to generate this figure are shown in Additional file 4: Table S3.

The location of palindromes in the unique portion of the genome can be observed as regions with a higher than expected number of sequence reads. Our estimated coverage of the non-repetitive sequences (~8 x 10^7^ bp) mapped to total unique genome sequences (~1.26 x 10^9^ bp) is ~6%. To determine palindrome locations, we looked for unique sequence regions that were over-represented as determined by the base read ratio “*B*”. For a single read mapped to the unique region of the genome, *B* = 1 (Figure 1B-1&2). For overlapping reads forming a contiguous genomic region (contig) “*C*”, the base read ratio is the sum of the read lengths divided by the length of the contig. Thus for contig “*c*”  based on the total length of uniquely mapped reads where *n(c)* is the number of reads in contig *c*, *ReadLength(r,c)* is the read length of read *r* in contig *c,* and *ContigLength(c)* is the length of contig *c.* Contigs are limited to the mapped unique sequences and exclude repetitive sequences masked by Repeat Masker. To combine adjacent contigs that are likely to represent a single locus, we joined contigs where *B* ≥ 1.5. For pragmatic reasons we focused on enriched unique sequence intervals that were within 7.5 kb from each other (Figure 1B-3). Enrichment of joined contigs was compared by using a Rank Score “*R*” calculated as the sum of the read lengths assigned to each joined contig divided by the length of the joined contig minus the length of the masked regions (*M)*, thus for JoinedContig “a”  (Figure 1B-4).

To demonstrate the stringency and specificity of our criteria, we conducted a simulation analysis to look for random hotspots based on our coverage (See Methods). The computer randomly selected positions for an equivalent number and bp of mapped reads in the genome and clustered them using the same parameters used in identifying our genuine palindrome candidates. In the simulated data 4% of reads fell into regions with base read ratio (*B*) >1.5. After clustering into joined contigs, we identified the top 50 loci, all of which had a rank (*R*) of <0.75 (Table 2). Using the same metrics, only 9 regions of IMR-90 (including 2 from Y chromosome) had *R* >0.75 (Table 2 and Additional file 1: Table S2). In contrast, 35 of the regions identified in MCF-7 had *R* >0.75 that can be considered good candidates for DNA palindromes (Additional file 2: Table S1). Two regions with highly clustered palindromes were identified on chromosomes 17 and 20, which are consistent with a high degree of rearrangement seen in these regions from previous studies [20, 22].

**Table 2:** **Summary of palindrome candidate data**

Rank	MCF7	IMR90	Random
0.05-0.75	130 (79%)	55 (86%)	46 (100%)
0.75-7.5	34 (21%)	8 (12.5%)	0
>7.5	1 (0.006%)	1 (1.5%)	0
Total	165	64	46

We also sequenced GAPF prepared DNA from MCF-7 and IMR90 by Illumina sequencing. In MCF-7, 25 million reads were generated with average length 36 bps, and 94% of the reads were mapped. The mapped bp was equivalent to ~28% of the total human genome (hg18). The Illumina sequencing data yielded a higher coverage of the genomic DNA and was used for evaluating Roche 454 identified palindrome candidates.

### Sorting of the palindrome candidates for physical analysis

For the 35 palindrome candidates obtained by the bioinformatic and statistical analyses, we further analyzed their read density by plotting the number of sequence reads in 1 kb bins extending over the enriched areas including 10 kb upstream and downstream (Additional file 3: Figure S1). Although the size of palindromes could be several Mbp in the genome, the genomic DNA isolation step shears the DNA into smaller fragments generally less than about 50 kb. In addition, denatured palindromes reanneal more efficiently in regions closer to the palindrome center. Therefore, the palindromic DNA closest to the center is more highly enriched than sequences further away. The result is a signature pattern represented by a higher read density toward the palindrome center. However, this pattern is obfuscated by problems associated with mapping repeated sequences. For example, a 1 kb bin that corresponds to a repeated sequence could be over represented because of the faster renaturation kinetics of repeated sequences, or it could be underrepresented if reads from repeated elements were removed by the algorithm used to map the reads (in our case Repeat Masker). Using the read density information, we examined our 35 MCF-7 palindrome candidates (Additional file 3: Figure S1) for the signature pattern. One candidate that exhibited this pattern in both the 454 and Illumina data corresponded to a palindrome previously (Chr8:128,202,704-128,210,979) identified [12]. We chose five additional candidates with this pattern and further characterized them by identifying the novel junctions associated with their formation and determining the spacer between the inverted repeats. The methods used for this analysis are illustrated for one of the candidates below.

### Mapping of the palindrome spacer and novel junction

The novel DNA junction created by palindrome formation may provide clues to the mechanism(s) by which they were formed. Since the S1 nuclease treatment in our protocol removes the hairpins and/or spacers of the palindromes, we have established new approaches to isolate the novel palindrome junctions.

#### Predicting the location of palindrome center

The read density signature described above predicts the center of a candidate palindrome as closest to the region with the highest number of reads. For example, the enriched central region of the chromosome 15q21.1 palindrome is about 21 kb (47,529,204-47,550,373). When plotting the read numbers for the 21 kb region of Chr15q21.1 plus 10 kb upstream and downstream, we found a gradient of reads in both the 454 and Illumina data, suggesting that the center of the palindrome is towards the centromere (Figure 2A).

The enrichment of the central region of the palindrome can also be detected by quantitative PCR. We used Taqman qPCR to compare the MCF-7 DNA palindrome candidates as compared to the same sequences from IMR90 DNA, or non-enriched unique sequences from MCF-7 DNA. Comparison of the Ct values (threshold cycles, the number of cycles at which the fluorescence detected exceeds the threshold, a relative measure of the amount of target DNA) before and after snap-back plus S1 digestion were used to calculate the amount of DNA protected in each sample. For example, we used a non-palindromic single copy gene, *RAD52,* as a control and found that the Ct value increased ~ 10–12 cycles for the primer set for *RAD52* in all DNA samples tested (Figure 2B and C). This corresponds to more than 1000-fold depletion of the DNA. In contrast, the primer pair P2 from the chromosome 15q21.1 palindrome candidate only had a 3 Ct cycle increase in MCF-7. This 7-cycle difference suggests a relative enrichment of P2 to RAD52 of over 100-fold for this region in MCF-7. This enrichment for the P2 target was not seen in the control cell line IMR-90, suggesting a *de novo* palindrome arose in the MCF-7. The P1 target, only ~1 kb centromere-proximal to P2, was not enriched indicating it was located outside of the palindrome region. We have also done qPCR using primer pairs P3 and P4, which are located on the palindrome but further away from the center. Those two primer pairs showed a similar, but somewhat lesser, relative enrichment as the P2 target, indicating that our Taqman qPCR approach can detect enrichment as distant as 10 kb from the palindrome center.

#### Analysis of palindrome structure

To further analyze the 15q21.1 palindrome structure in the genome, we used Southern blot analyses to monitor rearrangement associated with the palindrome. We chose restriction enzymes BamHI, BglII and NcoI to digest the genomic DNA because we expected to see novel bands with these enzymes in MCF-7 DNA (sites noted on Figure 2C). As predicted from the map in Figure 2C, we found rearranged bands (*) corresponding to the palindrome in MCF-7 that were not found in IMR-90 with the increasing size as expected (Figure 2D). Next we further analyzed BamHI-digested DNA by comparing untreated genomic DNA to melted and self-annealed (snap-back) treated DNA in MCF-7 and IMR-90. We found a half-sized fragment after snap-back treatment of MCF-7 DNA (Figure 2E, arrowhead, SB) indicating intra-strand reannealing, thus confirming the palindromic structure of the Chr15q21.1 candidate.

#### Inversion PCR to recover palindrome junctions

Defining the sequence at the novel junction of palindromes might provide mechanistic insights regarding how the palindromes are formed. To recover the novel junction, we developed a technique based on finding a spacer between the inverted repeats of the two palindrome arms (Figure 2F). By designing several oligonucleotides from the same strand we tested for PCR products with each other. If one oligonucleotide maps to the spacer and another to the inverted repeat, a PCR product will be formed due to the inverted repeat nature of a palindrome. The PCR product was cloned and the novel junction at the center of palindrome sequenced. By this approach, we identified a novel rearrangement that did not exist in the control IMR90 (Figure 2F). The spacer was a 467 bp and included a 185 bp insertion from Chr16 (Figure 3). We do not know if the original creation of the palindrome involved interactions with Chromosome 16, or whether this was a secondary event to stabilize a palindrome with a much shorter initial spacer. The 15q21.1 palindrome center is located in an intron of *FGF7* gene such that the palindrome contains duplications of exons 3 and 4 include the FGF signature motif and alpha 1,4 glycosyltranferase, respectively. It is possible that this rearrangement might have contributed to the tumorgenesis of MCF-7 breast cancer.

Using similar strategies, we confirmed four other palindromes in MCF-7 and determined their spacer and junction sequences derived from Chr15q21.3, Chr8q21.2, Chr1q31.3 and Chr20q13.2 (Table 3). An increase in spacer size correlated with a decrease in Rank Score, indicating that intra-strand annealing is more efficient when the spacer is smaller. A total of seven novel breakpoints were mapped and the junction sequences are shown in Figure 3. The microhomologies present at the junctions ranged from 0-7 bp, indicating that most of the junctions were probably made by non-homologous end joining (NHEJ). We also determined whether the seven novel junctions were present in five different sublines of MCF-7 cells (MCF-7-neo, MCF-7-BK, MCF-7-B, MCF-7-C and MCF-7-NCI60) [25] by using the same PCR primers used to identify the junctions in MCF-7 cell line we obtained from ATCC for this study. We confirmed that all seven novel junctions were present in MCF-7-neo, MCF-7-C, MCF-7-NCI60 and MCF-7 from ATCC; the six novel junctions from Chr15q21.1, Chr15q21.3, Chr8q21.2 and Chr20q13.2 were present in MCF-7-BK; the three junctions from Chr15q21.1 and Chr15q21.3 were present in MCF-7-B. Loss or gain of junctions in different cell lines could reflect continuing instability.

**Table 3:** **Summary of characterized MCF7 palindrome spacers**

Chromosome	Assembled 454 palindrome contigs	454 palindrome rank score	Spacer size (bp)	Spacer location	Insertion or deletion in Spacer
Chr15 (q21.1)	47,528,609-47,550,373	2.41	467	47,528,051-47,528,326	Insertion: Chr16(-):185 bp
					44,946,106-44,945,921
Chr15 (q21.3)	52,336,749-52,346,086	2.53	657	52,346,284-52,346,941	None
Chr8 (q21.2)	86,478,506-86,486,590	2.01	2286	86,417,409-86,478,509	Deletion: Chr8(+): 58,813 bp
					86,418,384-86,477,197
Chr1 (q31.3)	196,632,634-196,644,263	1.25	1555	196,630,725-196,632,280	None
Chr20 (q13.2)	51,618,339-51,633,176	0.83	430	51,615,731-51,616,160	None

### Comparison between GAP-Seq and microarray based GAPF method

The microarray-based GAPF approach has been used for detecting palindromes in cancer cells, and >80 GAPF positive cytogenetic bands were identified in MCF-7 [9, 28]. Subsequently, Diede et al. modified the GAPF approach by introducing 50% formamide in DNA denaturation step to remove false positive signals from non-palindromic regions that were found to correlate with regions of high DNA methylation [28]. Guenthoer et al. next re-examined GAPF profiles in MCF-7 breast cancer cell line as well as the control cell line IMR90. They found total 52 GAPF positive regions in MCF-7 and physically mapped one region on Chr8 (128,201,619-128,208,246) [12]. 39 of their GAPF positive regions were less than 1 kb and 7 were less than 100 bp. The authors recognized that identifying true palindromes remains elusive and pointed out two possibilities for false GAPF positives: 1) Repeat sequences in the genome, such as Alus, LINEs, or short tandem repeats, can obfuscate the identification of palindromes; 2) The limitations in the sensitivity of their approach cannot detect palindromes in a subpopulation of cells in heterogeneous tumor samples [12]. Their use of restriction enzyme digested DNA might limit the ability of palindrome detection.

The GAP-Seq approach significantly improves on the detection of true palindromes in several aspects: 1) The use of high-molecular weight DNA rather than enzyme digested DNA results in the recovery of longer sequences making identification more likely; 2) Read density distribution adds another feature characteristic of palindrome candidates (Additional file 3: Figure S1); 3) The read density distribution also provided us with important information about the orientation of the center of the palindrome, which was important in the isolation and sequencing of the palindrome junction and spacer. Using GAP-Seq we were able to identify and verify novel junctions that have never been reported in the plethora of previous studies of MCF-7 and provide an important extension to previous attempts to characterize this cell line.

### Examination of biological consequences associated with identified palindromes

To further understand the biological significance of the *de novo* palindromes in our analysis, we investigated the spatial association between palindromes and increased copy number in the MCF-7. Palindrome formation is an underlying mechanism of gene amplification, as it increases the copy-number from one to two [14]. We compared our data with Affymetrix SNP6 array data, and analyzed the correlation between our 35 palindrome candidates and copy number variation breakpoints (CNVB) (Figure 4 and Additional file 4: Table S3). We calculated the distance from either side of a palindrome candidate to its nearest CNVB. 8 of 35 palindrome candidates are located less than 5 kb from their nearest CNVB. These included the 6 confirmed palindrome candidates and two additional candidates (Chr7:113,925,138-113,935,162 and Chr13: 46,991,099-46,999,671). The remaining 27 candidates are located between several hundred Kb to several Mb away from the nearest detectable CNVB. Because this distance is dependent on the quality of both 454 and CGH data, it remains unclear whether they should be eliminated as good candidates for DNA palindromes. The association of palindrome candidates with CNVB will be useful to validate true palindromes. These data indicate a good correlation between palindrome candidates and gene amplification.

Some of the identified palindrome candidates were associated with amplified genomic regions that contain cancer genes. Cancer genes are defined as genes that when mutated are causally implicated in oncogenesis [34]. The confirmed palindrome in 8q24.21 (Chr8:128,202,704-128,210,979) was co-amplified with the *MYC* oncogene. Two palindrome candidates, 17q23.2 (Chr17: 56,691,822-56,700,625) and 20q13.2 (Chr20: 52,771,235-52,783,881), contained the *BCAS3 and ZNF217* genes that was amplified and overexpressed in breast cancers and was often associated with chromosomal alterations affecting the locus [35–37].

## Discussion

In this study, we developed a new strategy to detect DNA palindromes by coupling fast annealing genomic DNA treated by S1 nuclease (GAPF) with high-throughput sequencing (GAP-Seq) and recovery of novel palindrome junctions. We chose to use the MCF-7 breast cancer cell line for this initial proof-of-principle study because it has been extensively analyzed at the genomic level, allowing us to determine if our approach could generate novel data. In fact, none of our palindrome junctions had been identified by either sequence analysis or novel breakpoint analyses of MCF-7 [22, 25, 26]. This difference may be a result of either or both of two constraints presented by the characteristics of palindromes: 1) the breakpoint analysis was done from BAC clones, where palindromes are not stable during *E.coli* propagation, and 2) most of novel breakpoints identified here are located in or near to repeat-masked regions and would not be recovered by mapping of high-throughput sequencing data without knowing more about the sequences surrounding palindrome center. Therefore, palindromes are likely an underestimated structure of somatic rearrangements in cancer and other associated human diseases.

Although the palindrome junctions sequenced here have fairly large spacers and should theoretically be stable in BAC clones, it is not clear whether very long inverted repeats are well tolerated in *E. coli*. Some of the inverted repeats associated with the palindromes are likely to be megabases long, possible reflections of chromosomal breakage-fusion-bridge (BFB) cycles [38]. Although the palindrome would include the entire length of the CNV fragment, only the central ~20-40 kb could be recovered in our study due to DNA shearing. Furthermore, a study of complex genomic rearrangements consisting of intermixed duplications and triplications of genomic segments at both the *MECP2* and *PLP1* loci demonstrates that long inverted repeats with larger spacers can lead to genome rearrangements and contribute to local instability in the human genome [39].

We mapped 7 novel breakpoints that have a 0–7 bp microhomology at the junctions (Figure 3), suggesting that they were made by NHEJ. However, it is possible that the junctions were made independent of NHEJ. Palindromes can be created by template switching of replication forks through microhomology (FoSTeS- Fork-Stalling and Template Switching) by Microhomology Mediated Break Induced Replication (MMBIR) [40–43], or by foldback replication [13, 44]. The microhomologies identified at three of the sequenced junctions (Chr15q21.3 breakpoint 3, Chr8q21.2 breakpoint 4, and Chr1q31.1 breakpoint 6) could reflect a foldback priming mechanisms as seen in our previous analysis of palindromes from yeast [13]. However, it is difficult to determine if a similar mechanism is functioning with such a small data set. Two of the palindromes contained complex junctions including an insertion from another chromosome (Chr15q21.1) and the deletion of local sequences (Chr8q21.2). Such events could reflect more complicated pathways for their initial formation or secondary events indicative of the instability of the initial palindrome structure. The presence of two contiguous palindrome breakpoints on Chr15 leads us to speculate that there was an initial double strand break at the more telomere proximal site. This could have led to subsequent BFB cycles that may have generated further amplification and the second more centromere-proximal palindrome.

The palindrome candidates located in clusters on Chr17 and Chr20 all were contained within a large highly amplified region, indicating that secondary events might have resulted in more complicated genomic rearrangements at these loci. The complex genome amplification patterns seen in some breast cancers are characterized by multiple closely spaced amplicons, frequent high-level amplifications, and are highly correlated with aggressive disease [45]. These patterns are suggestive of a palindromic structure and associated genomic instability although this correlation has not been examined. Based on our observations, we hypothesize that an initial event might be the formation of a palindrome, which can then lead to genome instability and further amplification. This could provide a mechanism for amplifying cancer-associated genes, which are then selected for during cancer development.

## Conclusions

We have developed a new strategy to detect palindromes and recover their junctions in the genome. Our GAP-Seq approach improves upon previous microarray-based GAPF technique by combining GAPF with high-throughput sequencing. Our bioinformatics analysis also provides us with palindrome orientation information that is critical for junction recovery. Taken together, we show here that we can overcome the previous barriers due to the large number of false positives that obfuscate analysis of true palindromes. Using MCF-7 breast cancer cell line as the proof-of-principle analysis, we have identified 35 palindrome candidates and physically characterized the top 5 candidates and their junctions, proving that our strategy can correctly predict palindrome orientation and recovery of the novel DNA junctions associated with palindromes. Despite extensive analysis of MCF-7 at the molecular level, these data are novel and are missing from previous analyses of this cell line. Our approach underscores the importance of developing new tools for identifying and characterizing palindromes, and provides a new strategy to systematically identify palindromes in genomes.

## Methods

### Cell culture

The human breast cancer cell lines MCF-7 and IMR-90 (CCL-186) primary fibroblast were obtained from the American Type Culture Collection (ATCC). Cell lines were maintained under standard culture conditions (ATCC) and harvested at log phase.

### Roche 454 sequencing

The genomic DNA from cells was extracted by the Blood & Cell Culture DNA Kits (Qiagen) according to the manufacturer’s instructions. To prepare for a 454 sequencing library, the genomic DNA was denatured in the presence of 50% formamide and reannealed briefly in 100 mM NaCl on ice, then subsequently treated with S1 nuclease as previously described [28] with the following modifications: We started with ~100 μg of genomic DNA. After snap-back and S1 nuclease treatment, we twice extracted the DNA with UltraPure Phenol:Chloroform:Isoamyl Alcohol (25:24:1, v/v) (Invitrogen). The DNA was then precipitated in 100% ethanol, washed with 70% ethanol and dissolved in 1xTE buffer. We prepared Roche 454 libraries sheared to approximately 500 bp fragments and sequenced with the Roche 454 GS FLX + system by the standard method.

### Illumina sequencing

Deep sequencing of the 36-mers was obtained using Illumina Genome Analyzer IIx at the Ohio State University James Cancer Hospital. High molecular weight genomic DNA was obtained from MCF-7 and IMR-90. Cells were harvested and were incubated in the lysis buffer (100 mM NaCl/10 mM Tris·HCl, pH 8.0/25 mM EDTA/0.5% SDS/Proteinase K) for 24 hours at 37°C, followed by phenol/chloroform extraction and ethanol precipitation as described previously [9]. Briefly, one mg of genomic DNA was first digested with either restriction enzyme KpnI or SbfI. After heat-inactivation of restriction enzymes, both digests were pooled and denatured with formamide in boiling water for 7 minutes followed by quick renaturation on ice in 100 mM NaCl. Single-stranded DNA was digested by S1 nuclease at 37°C for 1 hour. Processed DNA samples were purified using the PCR-clean up kit (Promega). DNA was fragmented by sonication using a Covaris S2 and 200 bp DNA fragments were used for the construction of a sequencing library using the Illumina CHIP-SEQ kit.

### Affymetrix SNP6 copy number analysis

Genome-wide copy number analyses for MCF-7 and IMR-90 were performed using SNP6.0 (Affymetrix) at the Case Comprehensive Cancer Center (P30 CA43703). Two mg of genomic DNA was processed for hybridization using the SNP6 core reagent kit. The data were analyzed using Partek Genomics Suite (Partek). Raw data were normalized using the Robust Multi-Array Average (RMA) method. RMA consists of three steps: a background adjustment, quantile normalization [46] and final summarization. Normalized data were used to calculate the relative copy number of MCF-7 to IMR-90.

### Roche 454 data analysis

#### Mapping and content analysis

The 454 reads were masked with RepeatMasker [30]. The remainder of the sequences were mapped with BLAT [47] to the Human genome sequence (Version hg18, repeats masked) with condition of at least 75% identity of at least 40 bp. Sequences that were not mapped to the genome with these conditions were subjected to the metagenomic analysis pipeline (Smythers and Volfovsky; unpublished observations). This analysis identified additional matches to human DNA from GenBank, missing in previous hg18 analyses.

#### Random palindrome simulation analysis

The locations of mapped reads from MCF-7 were randomly assigned in the genome based on the actual number of reads and projected read lengths observed. When a repeat-masked region was encountered during simulation, the procedure was repeated in a new random location. The resulting null distribution data were clustered using the same parameters as we used to identify palindromes to generate a null simulation palindrome data set.

### Illumina sequencing data analysis

Illumina data were mapped with Bowtie to human reference genome (hg18) with the default module (-k 1). 35 GAP-Seq positive regions in MCF7 and 9 GAP-Seq positive regions in IMR90 from Roche 454 data analysis (R > 0.75) were binned as 1000 bp (Additional file 3: Figure S1 and Additional file 5: Figure S2).

### Real-time qPCR analysis of palindromes

Real-time qPCR was used to assess the enrichment of a palindrome over a single copy non-palindrome region (*RAD52*). We used TaqMan probes (Applied Biosystems) to genomic regions within the 454 positive signal. Real-time qPCR reactions used the Master mix from Applied Biosystems and were run according to manufacture’s instructions on a Bio-Rad multicolor Real-Time qPCR detection machine (IcyclerIQ) and analyzed with Icycler3.1 IQ software. Primer sequences are listed in Additional file 6: Table S4.

### PCR analysis of palindrome junctions

All PCR reactions were performed under standard conditions as recommended by the manufacturer (Clontech) using Titanium Taq polymerase. MCF-7 subline genomic DNAs (MCF-7-neo, MCF-7-BK, MCF-7-B, MCF-7-C) were obtained from Dr. Adrian Lee’s lab. To get the palindrome junctions, we designed PCR primers based on the unrearranged chromosome using Primer-Blast program on NCBI website [48]. The PCR products were cloned using TOPO TA cloning kit (PCR 2.1-TOPO, Invitrogen) and the cloned products were isolated and sequenced using Sanger sequencing (LMT, SAIC Frederick). Primer sequences are listed in Additional file 6: Table S4.

### Southern blotting

Southern blotting and snap-back southerns were performed as previously described [9].

### Availability of supporting data

The data sets supporting the results of this article are available in The Gene Expression Omnibus (GEO) with accession number GSE43679 and The NCBI Sequence Read Archive (SRA) with accession ID SRA064847 and SRA065361.

The data sets supporting the results of this article are included within the article and its additional files.

## Electronic supplementary material

Additional file 1: Table S2: List of palindrome candidates for IMR90. (DOCX 46 KB)

Additional file 2: Table S1: List of palindrome candidates for MCF7. (DOCX 86 KB)

Additional file 3: Figure S1: Read density analysis (1 kb-bin) for 35 GAP-Seq positive regions (R > 0.75) in MCF-7. (DOCX 3 MB)

Additional file 4: Table S3: Positions of MCF7 palindrome candidates and copy number changes. (DOCX 146 KB)

Additional file 5: Figure S2: Read density analysis (1 kb-bin) for 9 GAP-Seq positive regions (R > 0.75) in IMR90. (DOCX 636 KB)

Additional file 6: Table S4: Oligonucleotide sequences. (DOCX 87 KB)

## Abbreviations

BAC: Bacterial artificial chromosome
BFB: Breakage-fusion-bridge
bp: Base pair
CNVB: Copy number variation breakpoints
DSB: Double strand DNA break
GAPF: Genome-wide Analysis of Palindrome Formation
mtDNA: Mitochondria DNA
NHEJ: Non-homologous end joining
PATRR: Palindromic AT-rich repeats
qPCR: Real-Time quantitative PCR
GAP-Seq: GAPF technique with high-throughput sequencing.
