# Supplementary material for: GAP-Seq: a method for identification of DNA palindromes
Source: BMC Genomics. 2014 May 22;15(1):394. doi: 10.1186/1471-2164-15-394 (PMC4057610; doi:10.1186/1471-2164-15-394)
Supplement: Supplementary file 1 — Additional file 1: Table S2: List of palindrome candidates for IMR90. (DOCX 46 KB) [file 12864_2013_6105_MOESM1_ESM.docx]

**Supplemental Table 2. List of palindrome candidates for IMR90**

| **Chromosome** | **Start** | **End** | **Length (bp)** | **Rank Score** | **Gene** |
| --- | --- | --- | --- | --- | --- |
| 2 | 132,731,578 | 132,750,048 | 18,470 | 1.65 |  |
| 4 | 124,427,663 | 124,435,290 | 7,627 | 2.73 | *SPATA5* |
| 6 | 32,605,660 | 32,665,570 | 59,910 | 5.12 | *HLA-DRB1, HLA-DRB5* |
| 7 | 110,754,904 | 110,762,821 | 7,917 | 1.07 | *IMMP2L* |
| 16 | 33,857,643 | 33,868,362 | 10,719 | 2.32 |  |
| 17 | 42,567,848 | 42,621,659 | 53,811 | 50.29 | *CDC27* |
| 19 | 6,982,271 | 6,990,505 | 8,234 | 3.91 |  |
| Y | 57,422,793 | 57,434,454 | 11,661 | 5.96 |  |
| Y | 10,630,457 | 10,639,703 | 9,246 | 1.11 |  |
