# Supplementary material for: GAP-Seq: a method for identification of DNA palindromes
Source: BMC Genomics. 2014 May 22;15(1):394. doi: 10.1186/1471-2164-15-394 (PMC4057610; doi:10.1186/1471-2164-15-394)
Supplement: Supplementary file 2 — Additional file 2: Table S1: List of palindrome candidates for MCF7. (DOCX 86 KB) [file 12864_2013_6105_MOESM2_ESM.docx]

**Supplemental Table 1. List of palindrome candidates for MCF7**

| **Chromosome** | **Start** | **End** | **Length (bp)** | **Rank Score** | **Gene** |
| --- | --- | --- | --- | --- | --- |
| 1 | 107,488,815 | 107,497,205 | 8,390 | 0.88 | *NTNG1* |
| 1 | 196,632,634 | 196,644,263 | 11,629 | 1.25 |  |
| 2 | 132,731,674 | 132,751,240 | 19,566 | 11.96 |  |
| 3 | 63,987,909 | 63,995,677 | 7,768 | 0.97 |  |
| 3 | 171,871,651 | 171,879,592 | 7,941 | 1.91 |  |
| 6 | 153,293,135 | 153,300,979 | 7,844 | 1.46 |  |
| 7 | 69,176,688 | 69,186,514 | 9,826 | 0.98 | *AUTS2* |
| 7 | 113,925,138 | 113,935,162 | 10,024 | 1.14 | *FOXP2* |
| 8 | 86,478,506 | 86,486,590 | 8,084 | 2.01 |  |
| 8 | 128,202,704 | 128,210,979 | 8,275 | 1.57 |  |
| 9 | 1,140,693 | 1,153,705 | 13,012 | 0.76 |  |
| 13 | 46,991,099 | 46,999,671 | 8,572 | 0.89 |  |
| 15 | 47,529,204 | 47,550,373 | 21,169 | 2.41 | *C15orf33,FGF7* |
| 15 | 52,336,749 | 52,346,086 | 9,337 | 2.53 | *UNC13C* |
| 16 | 33,860,065 | 33,868,823 | 8,758 | 2.03 |  |
| 17 | 54,411,568 | 54,420,717 | 9,149 | 0.92 | *PPM1E, TRIM37* |
| 17 | 56,691,822 | 56,700,625 | 8,803 | 1.27 | *BCAS3* |
| 17 | 56,835,678 | 56,848,467 | 12,789 | 0.83 | *C17orf82,TBX2* |
| 17 | 57,042,940 | 57,051,625 | 8,685 | 0.84 |  |
| 17 | 57,188,310 | 57,199,195 | 10,885 | 2.25 | *BRIP1* |
| 17 | 57,208,422 | 57,218,266 | 9,844 | 1.00 | *BRIP1* |
| 17 | 57,231,024 | 57,255,393 | 24,369 | 0.90 | *BRIP1* |
| 17 | 57,279,399 | 57,301,585 | 22,186 | 1.06 | *INTS2,BRIP1* |
| 20 | 45,338,173 | 45,353,804 | 15,631 | 0.88 |  |
| 20 | 45,411,773 | 45,420,743 | 8,970 | 0.80 |  |
| 20 | 51,618,339 | 51,633,176 | 14,837 | 0.83 |  |
| 20 | 51,659,015 | 51,672,527 | 13,512 | 1.25 |  |
| 20 | 51,692,525 | 51,711,906 | 19,381 | 1.03 |  |
| 20 | 51,775,306 | 51,790,907 | 15,601 | 0.99 |  |
| 20 | 51,907,065 | 51,925,914 | 18,849 | 0.97 |  |
| 20 | 51,965,461 | 51,980,596 | 15,135 | 0.89 |  |
| 20 | 52,037,921 | 52,054,026 | 16,105 | 1.16 | *BCAS1* |
| 20 | 52,086,640 | 52,094,418 | 7,778 | 0.94 | *BCAS1* |
| 20 | 52,599,634 | 52,610,577 | 10,943 | 0.92 | *DOK5* |
| 20 | 52,771,235 | 52,783,881 | 12,646 | 1.48 | *ZNF217* |
