# Supplementary material for: GAP-Seq: a method for identification of DNA palindromes
Source: BMC Genomics. 2014 May 22;15(1):394. doi: 10.1186/1471-2164-15-394 (PMC4057610; doi:10.1186/1471-2164-15-394)

Supplementary Figure 1. Read density analysis (1kb-bin) for 35 GAP-Seq positive regions (R>0.75) in MCF-7

**A) 6 GAP-Seq positive regions confirmed as palindromes with a read density signature:**


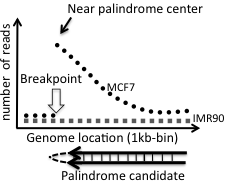
 OR
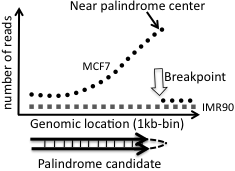


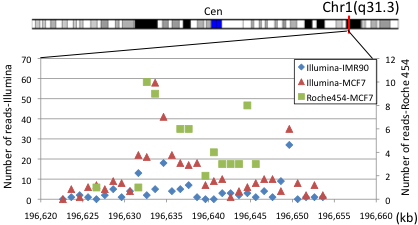

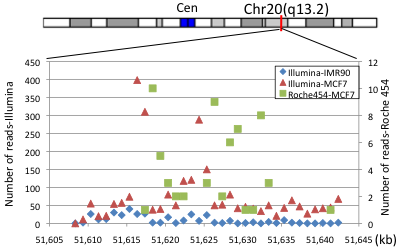


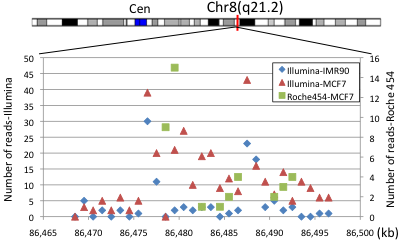

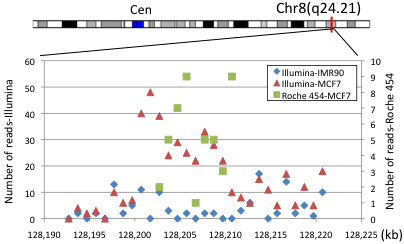


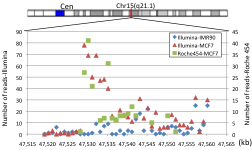

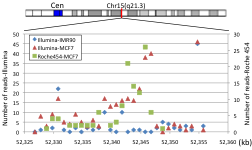


**B). Read density analysis for the other 29 GAP-Seq positive regions in MCF-7**


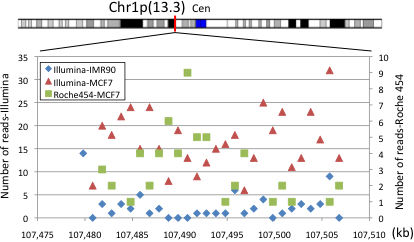

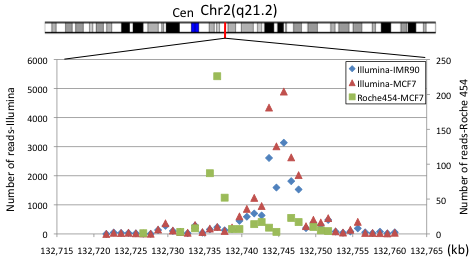


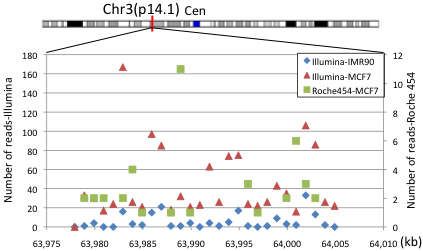

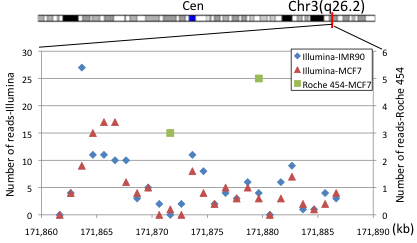


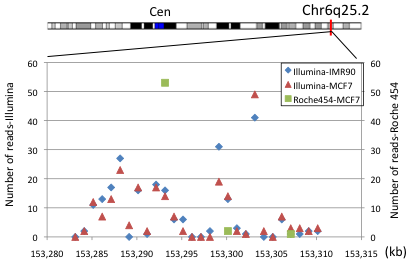

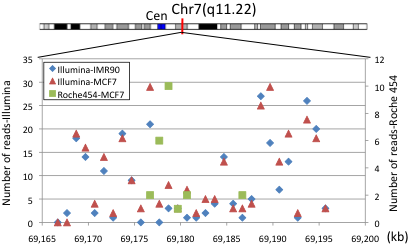


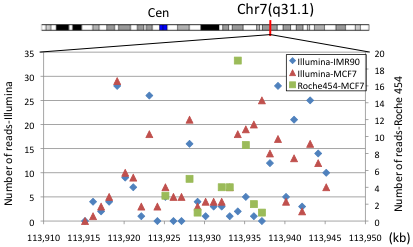

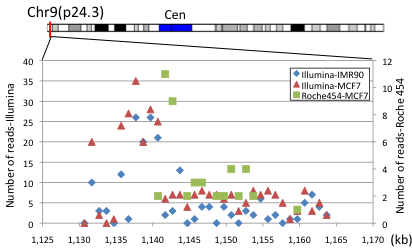


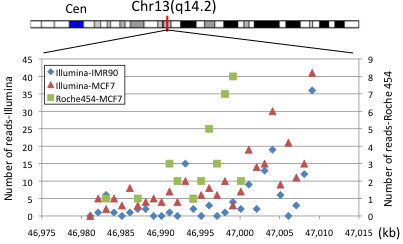

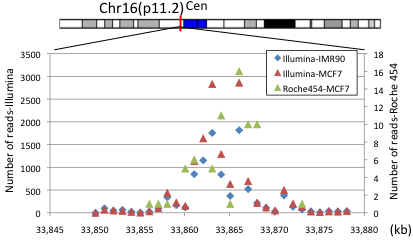

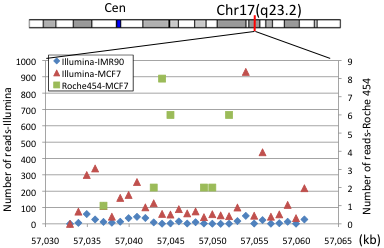

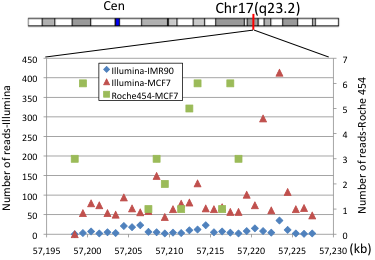

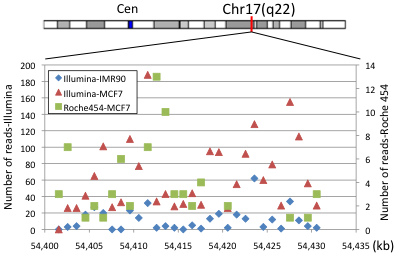

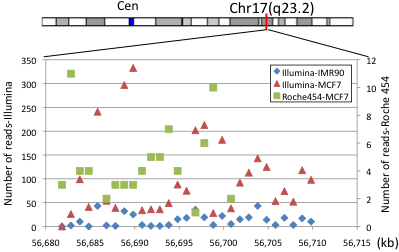


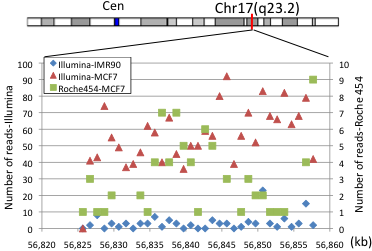

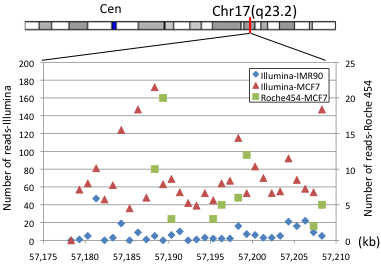


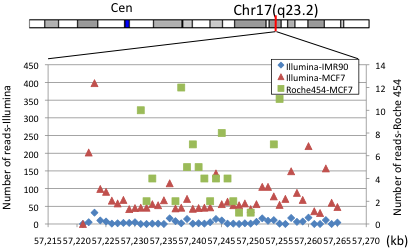

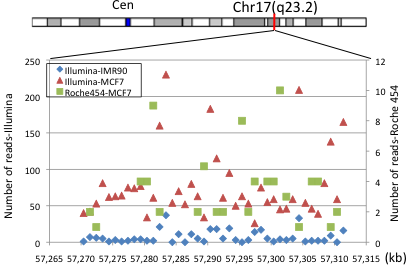


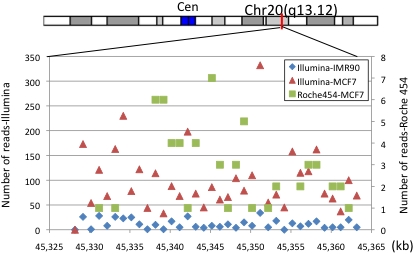

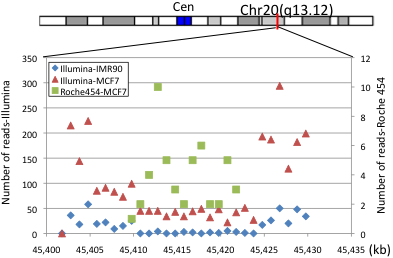


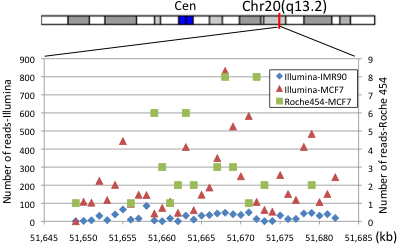

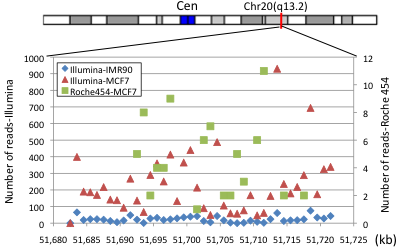


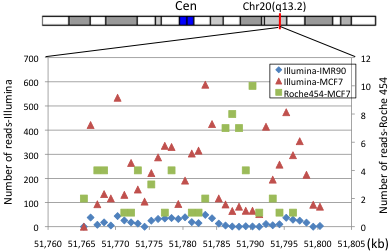

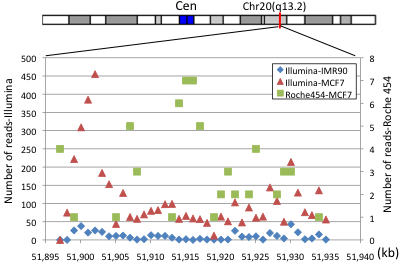


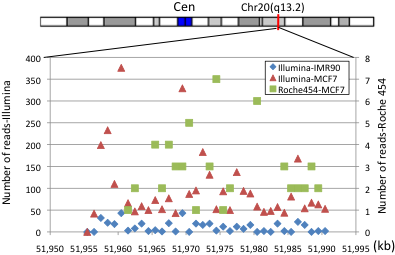

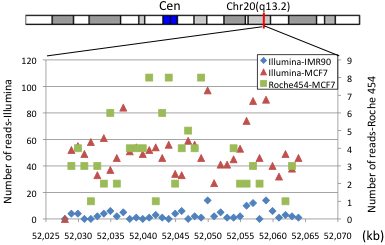


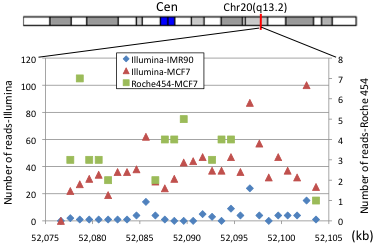

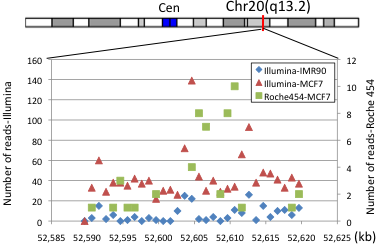


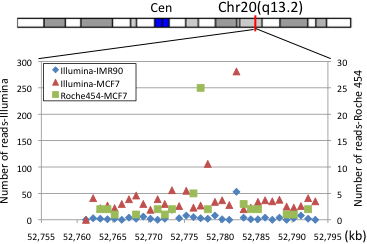

Supplement: Supplementary file 3 — Additional file 3: Figure S1: Read density analysis (1 kb-bin) for 35 GAP-Seq positive regions (R > 0.75) in MCF-7. (DOCX 3 MB) [file 12864_2013_6105_MOESM3_ESM.docx]
