# Supplementary material for: GAP-Seq: a method for identification of DNA palindromes
Source: BMC Genomics. 2014 May 22;15(1):394. doi: 10.1186/1471-2164-15-394 (PMC4057610; doi:10.1186/1471-2164-15-394)
Supplement: Supplementary file 4 — Additional file 4: Table S3: Positions of MCF7 palindrome candidates and copy number changes. (DOCX 146 KB) [file 12864_2013_6105_MOESM4_ESM.docx]

**Supplemental Table 3: Positions of MCF7 palindrome candidates and copy number changes**

|  | Roche 454 palindrome candidates |
| --- | --- |
|  | CGH array data |

| **Chromosome** | **Start** | **End** | **Length (bp)** | **Rank Score** | **Distance to CNV (bp)** | **Copy number** |
| --- | --- | --- | --- | --- | --- | --- |
| 1 | 72,541,006 | 72,583,736 | 42,730 |  |  | 5 |
| 1 | 106,537,258 | 107,731,728 | 1,194,470 |  |  | 5 |
| 1 | 107,488,815 | 107,497,205 | 8,390 | 0.88 | 234,523 (right) |  |
| 1 | 107,732,683 | 108,444,949 | 712,266 |  |  | 3 |
| 1 | 108,448,080 | 108,731,500 | 283,420 |  |  | 4 |
| 1 | 108,736,699 | 110,023,250 | 1,286,551 |  |  | 3 |
| 1 | 110,025,907 | 110,044,476 | 18,569 |  |  | 5 |
| 1 | 110,046,521 | 110,339,341 | 292,820 |  |  | 3 |
| 1 | 111,834,808 | 112,166,539 | 331,731 |  |  | 3 |
| 1 | 112,167,357 | 112,376,555 | 209,198 |  |  | 5 |
| 1 | 113,851,239 | 115,447,967 | 1,596,728 |  |  | 5 |
| 1 | 115,451,593 | 116,575,193 | 1,123,600 |  |  | 3 |
| 1 | 116,575,568 | 116,943,111 | 367,543 |  |  | 4 |
| 1 | 116,944,744 | 118,255,346 | 1,310,602 |  |  | 3 |
| 1 | 120,071,416 | 120,492,644 | 421,228 |  |  | 3 |
| 1 | 142,693,888 | 147,220,620 | 4,526,732 |  |  | 4 |
| 1 | 148,015,166 | 150,708,936 | 2,693,770 |  |  | 5 |
| 1 | 151,103,498 | 155,489,607 | 4,386,109 |  |  | 3 |
| 1 | 156,357,275 | 156,676,586 | 319,311 |  |  | 3 |
| 1 | 166,623,840 | 176,318,133 | 9,694,293 |  |  | 3 |
| 1 | 177,178,439 | 178,905,094 | 1,726,655 |  |  | 3 |
| 1 | 178,907,264 | 179,880,402 | 973,138 |  |  | 4 |
| 1 | 180,647,735 | 196,629,230 | 15,981,495 |  |  | 3 |
| 1 | 196,630,146 | 199,996,533 | 3,366,387 |  |  | 4 |
| 1 | 196,632,634 | 196,644,263 | 11,629 | 1.25 | 2,488 (left) |  |
| 1 | 200,253,607 | 205,978,228 | 5,724,621 |  |  | 4 |
| 1 | 212,880,110 | 217,836,841 | 4,956,731 |  |  | 4 |
| 2 | 43,277,606 | 43,591,677 | 314,071 |  |  | 3 |
| 2 | 80,917,132 | 105,884,418 | 24,967,286 |  |  | 3 |
| 2 | 108,357,112 | 117,649,493 | 9,292,381 |  |  | 3 |
| 2 | 119,768,167 | 122,388,887 | 2,620,720 |  |  | 3 |
| 2 | 124,289,666 | 135,398,851 | 11,109,185 |  |  | 3 |
| 2 | 132,731,674 | 132,751,240 | 19,566 | 11.96 | 2,647,611(right) |  |
| 3 | 35,345 | 6,395,009 | 6,359,664 |  |  | 3 |
| 3 | 6,630,078 | 10,344,524 | 3,714,446 |  |  | 3 |
| 3 | 11,038,174 | 13,656,391 | 2,618,217 |  |  | 3 |
| 3 | 13,658,626 | 23,477,214 | 9,818,588 |  |  | 3 |
| 3 | 61,580,689 | 64,923,168 | 3,342,479 |  |  | 5 |
| 3 | 63,987,909 | 63,995,677 | 7,768 | 0.97 | 927,491 (right) |  |
| 3 | 83,117,947 | 83,960,374 | 842,427 |  |  | 3 |
| 3 | 87,060,158 | 88,731,085 | 1,670,927 |  |  | 3 |
| 3 | 95,239,422 | 96,369,377 | 1,129,955 |  |  | 3 |
| 3 | 114,799,073 | 115,017,546 | 218,473 |  |  | 3 |
| 3 | 137,049,173 | 137,665,665 | 616,492 |  |  | 3 |
| 3 | 147,291,575 | 159,496,844 | 12,205,269 |  |  | 3 |
| 3 | 162,229,862 | 162,938,666 | 708,804 |  |  | 4 |
| 3 | 162,942,710 | 163,995,433 | 1,052,723 |  |  | 3 |
| 3 | 163,995,786 | 164,108,689 | 112,903 |  |  | 5 |
| 3 | 164,109,266 | 176,406,220 | 12,296,954 |  |  | 3 |
| 3 | 171,871,651 | 171,879,592 | 7,941 | 1.91 | 4,526,943 (right) |  |
| 3 | 176,406,535 | 176,882,478 | 475,943 |  |  | 5 |
| 3 | 176,894,618 | 178,300,248 | 1,405,630 |  |  | 3 |
| 3 | 192,440,852 | 196,497,615 | 4,056,763 |  |  | 3 |
| 3 | 196,497,953 | 199,380,515 | 2,882,562 |  |  | 4 |
| 4 | 70,162,233 | 70,266,388 | 104,155 |  |  | 4 |
| 4 | 107,046,743 | 107,294,920 | 248,177 |  |  | 4 |
| 4 | 143,728,081 | 144,632,048 | 903,967 |  |  | 3 |
| 5 | 68,532 | 1,728,417 | 1,659,885 |  |  | 3 |
| 5 | 10,909,634 | 11,289,536 | 379,902 |  |  | 3 |
| 5 | 14,239,651 | 14,919,969 | 680,318 |  |  | 3 |
| 5 | 77,125,709 | 96,936,826 | 19,811,117 |  |  | 3 |
| 5 | 96,943,590 | 102,138,572 | 5,194,982 |  |  | 3 |
| 5 | 102,146,391 | 112,577,634 | 10,431,243 |  |  | 3 |
| 5 | 112,919,768 | 116,130,853 | 3,211,085 |  |  | 3 |
| 5 | 116,136,300 | 134,500,866 | 18,364,566 |  |  | 3 |
| 5 | 135,276,633 | 154,638,210 | 19,361,577 |  |  | 3 |
| 5 | 155,427,831 | 158,794,816 | 3,366,985 |  |  | 3 |
| 5 | 160,404,092 | 166,655,123 | 6,251,031 |  |  | 3 |
| 5 | 167,553,902 | 177,413,869 | 9,859,967 |  |  | 3 |
| 5 | 177,415,125 | 177,520,625 | 105,500 |  |  | 5 |
| 5 | 177,530,548 | 180,722,926 | 3,192,378 |  |  | 3 |
| 6 | 94,661 | 716,987 | 622,326 |  |  | 3 |
| 6 | 721,120 | 5,132,644 | 4,411,524 |  |  | 3 |
| 6 | 5,336,267 | 29,395,268 | 24,059,001 |  |  | 3 |
| 6 | 30,048,351 | 57,314,189 | 27,265,838 |  |  | 3 |
| 6 | 153,293,135 | 153,300,979 | 7,844 | 1.46 | 95,978,946 (left) |  |
| 7 | 52,911 | 9,208,138 | 9,155,227 |  |  | 3 |
| 7 | 31,618,853 | 35,250,129 | 3,631,276 |  |  | 3 |
| 7 | 69,176,688 | 69,186,514 | 9,826 | 0.98 | 907,295 (right) |  |
| 7 | 70,093,809 | 77,710,003 | 7,616,194 |  |  | 3 |
| 7 | 77,712,011 | 100,200,945 | 22,488,934 |  |  | 4 |
| 7 | 100,203,345 | 109,221,034 | 9,017,689 |  |  | 5 |
| 7 | 109,241,218 | 113,935,238 | 4,694,020 |  |  | 5 |
| 7 | 113,925,138 | 113,935,162 | 10,024 | 1.14 | 76 (right), 3698 (right) |  |
| 7 | 113,938,860 | 128,390,528 | 14,451,668 |  |  | 3 |
| 7 | 128,396,866 | 131,199,493 | 2,802,627 |  |  | 3 |
| 7 | 132,335,038 | 135,143,264 | 2,808,226 |  |  | 3 |
| 7 | 136,672,025 | 141,492,075 | 4,820,050 |  |  | 3 |
| 7 | 142,156,294 | 142,167,486 | 11,192 |  |  | 5 |
| 7 | 142,176,272 | 143,321,436 | 1,145,164 |  |  | 3 |
| 7 | 143,995,941 | 148,381,489 | 4,385,548 |  |  | 3 |
| 7 | 148,404,842 | 148,565,600 | 160,758 |  |  | 5 |
| 7 | 148,567,024 | 157,927,938 | 9,360,914 |  |  | 3 |
| 7 | 157,930,384 | 158,329,612 | 399,228 |  |  | 5 |
| 7 | 158,338,824 | 158,819,765 | 480,941 |  |  | 3 |
| 8 | 76,708,988 | 86,479,904 | 9,770,916 |  |  | 3 |
| 8 | 86,478,506 | 86,486,590 | 8,084 | 2.01 | 1,398 (left),5,062 (right) |  |
| 8 | 86,481,528 | 91,766,339 | 5,284,811 |  |  | 5 |
| 8 | 91,774,589 | 100,987,006 | 9,212,417 |  |  | 3 |
| 8 | 100,991,549 | 104,787,418 | 3,795,869 |  |  | 4 |
| 8 | 104,787,498 | 107,128,543 | 2,341,045 |  |  | 5 |
| 8 | 107,131,499 | 110,411,775 | 3,280,276 |  |  | 4 |
| 8 | 110,415,463 | 112,433,955 | 2,018,492 |  |  | 5 |
| 8 | 112,439,129 | 121,625,944 | 9,186,815 |  |  | 3 |
| 8 | 121,626,368 | 127,876,972 | 6,250,604 |  |  | 5 |
| 8 | 127,888,705 | 128,206,914 | 318,209 |  |  | 3 |
| 8 | 128,202,704 | 128,210,979 | 8,275 | 1.57 | 4,210 (left), 1,430 (right) |  |
| 8 | 128,209,549 | 129,972,578 | 1,763,029 |  |  | 5 |
| 8 | 129,972,800 | 138,719,153 | 8,746,353 |  |  | 3 |
| 8 | 139,577,000 | 140,589,040 | 1,012,040 |  |  | 3 |
| 8 | 140,796,555 | 146,268,959 | 5,472,404 |  |  | 3 |
| 9 | 639,866 | 1,041,045 | 401,179 |  |  | 3 |
| 9 | 1,140,693 | 1,153,705 | 13,012 | 0.76 | 99,648 (left) |  |
| 9 | 105,087,487 | 105,890,672 | 803,185 |  |  | 3 |
| 10 | 62,759 | 11,728,482 | 11,665,723 |  |  | 3 |
| 10 | 13,572,654 | 13,694,808 | 122,154 |  |  | 4 |
| 11 | 5,746,177 | 5,765,715 | 19,538 |  |  | 5 |
| 11 | 45,752,246 | 50,725,893 | 4,973,647 |  |  | 3 |
| 11 | 64,947,784 | 80,595,403 | 15,647,619 |  |  | 3 |
| 11 | 81,017,369 | 86,242,992 | 5,225,623 |  |  | 3 |
| 11 | 117,203,693 | 118,481,929 | 1,278,236 |  |  | 3 |
| 12 | 32,930,369 | 34,402,050 | 1,471,681 |  |  | 3 |
| 12 | 34,467,977 | 38,610,920 | 4,142,943 |  |  | 3 |
| 12 | 38,689,043 | 45,394,930 | 6,705,887 |  |  | 3 |
| 12 | 45,395,084 | 52,708,787 | 7,313,703 |  |  | 3 |
| 12 | 60,456,215 | 64,521,079 | 4,064,864 |  |  | 3 |
| 12 | 64,521,731 | 65,012,706 | 490,975 |  |  | 4 |
| 12 | 65,013,893 | 97,824,594 | 32,810,701 |  |  | 3 |
| 12 | 98,163,860 | 108,929,121 | 10,765,261 |  |  | 3 |
| 12 | 119,107,564 | 119,465,854 | 358,290 |  |  | 3 |
| 12 | 131,231,646 | 131,699,734 | 468,088 |  |  | 3 |
| 13 | 18,469,629 | 45,492,023 | 27,022,394 |  |  | 3 |
| 13 | 45,502,754 | 45,929,714 | 426,960 |  |  | 4 |
| 13 | 45,945,213 | 47,002,795 | 1,057,582 |  |  | 5 |
| 13 | 46,991,099 | 46,999,671 | 8,572 | 0.89 | 3,124 (right) |  |
| 14 | 30,095,033 | 37,101,559 | 7,006,526 |  |  | 3 |
| 14 | 37,102,264 | 37,252,032 | 149,768 |  |  | 5 |
| 14 | 37,254,304 | 59,757,166 | 22,502,862 |  |  | 3 |
| 14 | 59,757,776 | 73,065,527 | 13,307,751 |  |  | 3 |
| 14 | 73,099,668 | 106,356,482 | 33,256,814 |  |  | 3 |
| 15 | 47,529,204 | 47,550,373 | 21,169 | 2.41 | 321 (left) |  |
| 15 | 47,529,525 | 52,339,254 | 4,809,729 |  |  | 5 |
| 15 | 52,336,749 | 52,346,086 | 9,337 | 2.53 | 2,505(left),1,019(right) |  |
| 15 | 52,347,105 | 54,846,324 | 2,499,219 |  |  | 3 |
| 15 | 60,812,237 | 74,665,417 | 13,853,180 |  |  | 3 |
| 15 | 74,689,234 | 100,286,563 | 25,597,329 |  |  | 3 |
| 16 | 7,902,449 | 8,095,495 | 193,046 |  |  | 3 |
| 16 | 16,077,978 | 21,501,135 | 5,423,157 |  |  | 3 |
| 16 | 21,853,559 | 31,697,991 | 9,844,432 |  |  | 3 |
| 16 | 33,860,065 | 33,868,823 | 8,758 | 2.03 | 2,162,074 (right) |  |
| 16 | 54,353,890 | 54,379,945 | 26,055 |  |  | 3 |
| 16 | 69,403,572 | 73,427,295 | 4,023,723 |  |  | 3 |
| 16 | 73,433,399 | 74,412,976 | 979,577 |  |  | 4 |
| 16 | 74,424,833 | 76,174,370 | 1,749,537 |  |  | 3 |
| 16 | 76,174,457 | 76,675,747 | 501,290 |  |  | 4 |
| 16 | 76,679,632 | 76,929,109 | 249,477 |  |  | 3 |
| 16 | 76,929,941 | 76,942,266 | 12,325 |  |  | 5 |
| 16 | 77,050,326 | 79,561,808 | 2,511,482 |  |  | 3 |
| 16 | 79,564,259 | 83,505,811 | 3,941,552 |  |  | 3 |
| 16 | 83,505,827 | 83,990,238 | 484,411 |  |  | 4 |
| 16 | 83,990,252 | 85,931,713 | 1,941,461 |  |  | 3 |
| 16 | 85,932,146 | 88,815,036 | 2,882,890 |  |  | 4 |
| 17 | 51,263,334 | 53,987,639 | 2,724,305 |  |  | 3 |
| 17 | 53,988,374 | 57,340,656 | 3,352,282 |  |  | 5 |
| 17 | 54,411,568 | 54,420,717 | 9,149 | 0.92 | 423,194 (right) |  |
| 17 | 56,691,822 | 56,700,625 | 8,803 | 1.27 |  |  |
| 17 | 56,835,678 | 56,848,467 | 12,789 | 0.83 |  |  |
| 17 | 57,042,940 | 57,051,625 | 8,685 | 0.84 |  |  |
| 17 | 57,188,310 | 57,199,195 | 10,885 | 2.25 |  |  |
| 17 | 57,208,422 | 57,218,266 | 9,844 | 1.00 |  |  |
| 17 | 57,231,024 | 57,255,393 | 24,369 | 0.90 |  |  |
| 17 | 57,279,399 | 57,301,585 | 22,186 | 1.06 | 39,071 (left) |  |
| 17 | 57,342,063 | 58,895,659 | 1,553,596 |  |  | 4 |
| 17 | 58,903,970 | 59,738,976 | 835,006 |  |  | 3 |
| 17 | 59,739,744 | 59,782,638 | 42,894 |  |  | 5 |
| 17 | 59,782,864 | 62,583,000 | 2,800,136 |  |  | 3 |
| 17 | 62,585,262 | 63,800,636 | 1,215,374 |  |  | 4 |
| 17 | 63,800,835 | 67,012,769 | 3,211,934 |  |  | 3 |
| 17 | 67,601,748 | 67,804,357 | 202,609 |  |  | 3 |
| 17 | 70,800,526 | 71,325,848 | 525,322 |  |  | 3 |
| 17 | 78,506,194 | 78,643,088 | 136,894 |  |  | 3 |
| 18 | 277,745 | 1,895,358 | 1,617,613 |  |  | 3 |
| 18 | 2,288,724 | 3,200,634 | 911,910 |  |  | 3 |
| 18 | 3,202,649 | 3,275,657 | 73,008 |  |  | 5 |
| 18 | 3,277,049 | 3,889,729 | 612,680 |  |  | 3 |
| 18 | 7,995,007 | 8,614,151 | 619,144 |  |  | 3 |
| 18 | 9,289,538 | 10,320,138 | 1,030,600 |  |  | 3 |
| 18 | 11,352,587 | 13,527,419 | 2,174,832 |  |  | 3 |
| 18 | 18,768,295 | 27,181,505 | 8,413,210 |  |  | 3 |
| 18 | 41,390,772 | 41,561,453 | 170,681 |  |  | 3 |
| 18 | 41,566,366 | 41,652,916 | 86,550 |  |  | 5 |
| 18 | 41,663,680 | 43,135,584 | 1,471,904 |  |  | 3 |
| 18 | 43,691,851 | 45,652,057 | 1,960,206 |  |  | 3 |
| 18 | 58,515,799 | 59,823,590 | 1,307,791 |  |  | 3 |
| 19 | 12,637,302 | 13,015,145 | 377,843 |  |  | 3 |
| 20 | 13,252,816 | 14,230,794 | 977,978 |  |  | 3 |
| 20 | 15,629,440 | 18,735,278 | 3,105,838 |  |  | 3 |
| 20 | 19,272,229 | 30,284,237 | 11,012,008 |  |  | 3 |
| 20 | 38,928,181 | 40,318,090 | 1,389,909 |  |  | 4 |
| 20 | 40,319,262 | 40,508,919 | 189,657 |  |  | 3 |
| 20 | 40,510,165 | 40,741,296 | 231,131 |  |  | 5 |
| 20 | 41,643,972 | 42,503,345 | 859,373 |  |  | 3 |
| 20 | 45,203,652 | 45,204,911 | 1,259 |  |  | 5 |
| 20 | 45,231,046 | 46,970,431 | 1,739,385 |  |  | 5 |
| 20 | 45,338,173 | 45,353,804 | 15,631 | 0.88 | 107,127 (left) |  |
| 20 | 45,411,773 | 45,420,743 | 8,970 | 0.80 | 1,929,644 (right) |  |
| 20 | 47,350,387 | 48,713,087 | 1,362,700 |  |  | 3 |
| 20 | 48,720,528 | 48,858,865 | 138,337 |  |  | 5 |
| 20 | 48,867,186 | 49,680,237 | 813,051 |  |  | 3 |
| 20 | 49,681,169 | 50,107,933 | 426,764 |  |  | 5 |
| 20 | 51,477,639 | 51,614,171 | 136,532 |  |  | 3 |
| 20 | 51,615,163 | 52,990,657 | 1,375,494 |  |  | 5 |
| 20 | 51,618,339 | 51,633,176 | 14,837 | 0.83 | 3,176 (left) |  |
| 20 | 51,659,015 | 51,672,527 | 13,512 | 1.25 |  |  |
| 20 | 51,692,525 | 51,711,906 | 19,381 | 1.03 |  |  |
| 20 | 51,775,306 | 51,790,907 | 15,601 | 0.99 |  |  |
| 20 | 51,907,065 | 51,925,914 | 18,849 | 0.97 |  |  |
| 20 | 51,965,461 | 51,980,596 | 15,135 | 0.89 |  |  |
| 20 | 52,037,921 | 52,054,026 | 16,105 | 1.16 |  |  |
| 20 | 52,086,640 | 52,094,418 | 7,778 | 0.94 |  |  |
| 20 | 52,599,634 | 52,610,577 | 10,943 | 0.92 | 380,080 (right) |  |
| 20 | 52,771,235 | 52,783,881 | 12,646 | 1.48 | 206,776 (right) |  |
| 20 | 52,991,500 | 53,583,567 | 592,067 |  |  | 4 |
| 20 | 53,584,595 | 53,599,493 | 14,898 |  |  | 5 |
| 20 | 53,599,885 | 55,096,040 | 1,496,155 |  |  | 3 |
| 20 | 55,097,277 | 55,285,876 | 188,599 |  |  | 5 |
| 20 | 55,291,318 | 56,391,714 | 1,100,396 |  |  | 3 |
| 20 | 56,395,042 | 56,990,364 | 595,322 |  |  | 4 |
| 20 | 56,991,047 | 58,998,640 | 2,007,593 |  |  | 3 |
| 20 | 59,235,553 | 59,537,363 | 301,810 |  |  | 3 |
| 21 | 9,758,742 | 10,030,151 | 271,409 |  |  | 3 |
| 21 | 35,112,470 | 35,156,070 | 43,600 |  |  | 3 |
| 21 | 38,303,965 | 46,921,385 | 8,617,420 |  |  | 3 |
| 22 | 33,087,576 | 33,390,353 | 302,777 |  |  | 3 |
