# Supplementary figures and images for: GAP-Seq: a method for identification of DNA palindromes
Source: BMC Genomics. 2014 May 22;15(1):394. doi: 10.1186/1471-2164-15-394 (PMC4057610; doi:10.1186/1471-2164-15-394)

Supplementary Figure 2. Read density analysis (1kb-bin) for 9 GAP-Seq positive regions (R>0.75) in IMR90


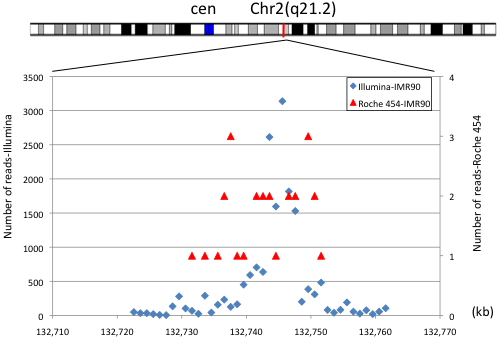

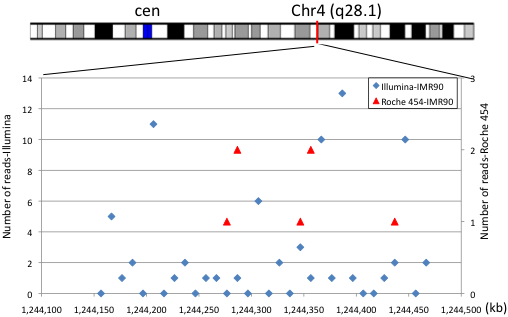


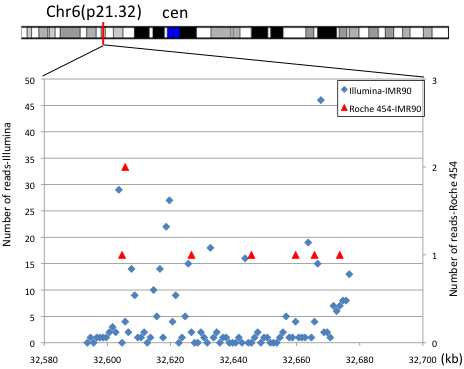

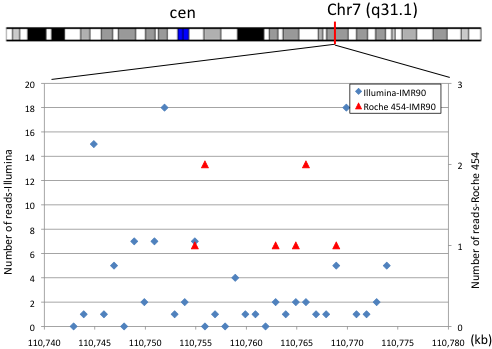


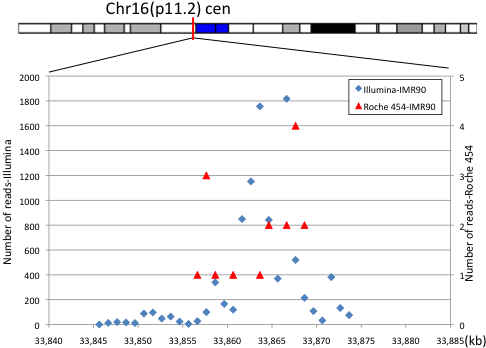

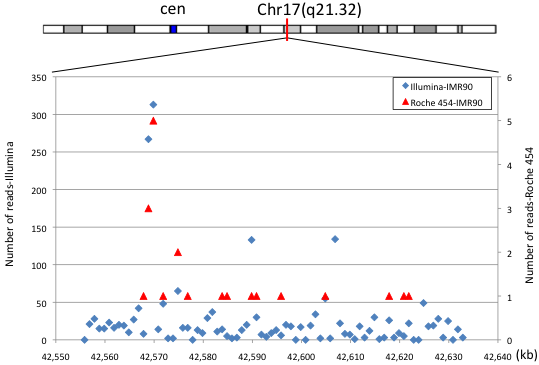


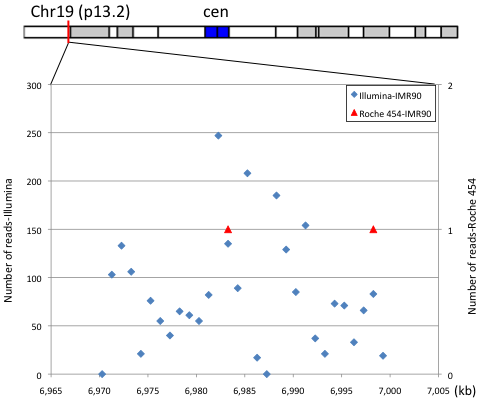

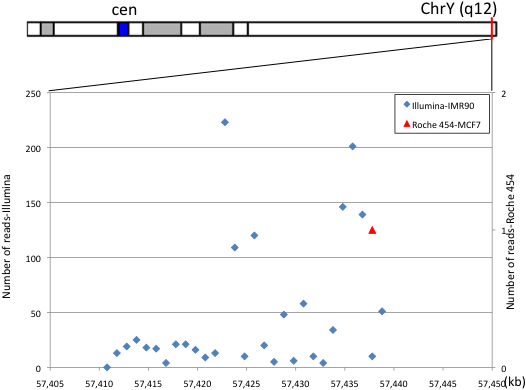


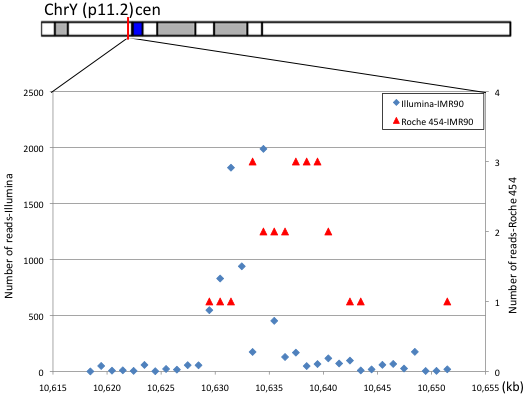

Supplement: Supplementary file 5 — Additional file 5: Figure S2: Read density analysis (1 kb-bin) for 9 GAP-Seq positive regions (R > 0.75) in IMR90. (DOCX 636 KB) [file 12864_2013_6105_MOESM5_ESM.docx]
