# Supplementary material for: GAP-Seq: a method for identification of DNA palindromes
Source: BMC Genomics. 2014 May 22;15(1):394. doi: 10.1186/1471-2164-15-394 (PMC4057610; doi:10.1186/1471-2164-15-394)
Supplement: Supplementary file 6 — Additional file 6: Table S4: Oligonucleotide sequences. (DOCX 87 KB) [file 12864_2013_6105_MOESM6_ESM.docx]

| Primer name | 5'--->3' |
| --- | --- |
| P1-FW | TGCAACCAGAGAAGAACTTCATCAT |
| P1-RV | GCTCTCGGACATTAAAATAATTTCAAACTCT |
| P1-FAM reporter | TCTGGAGCAGCTTTTG |
| P2-FW | ATGCCTTCTTACAGGGAAATTCACT |
| P2-RV | AGAAACTAAATTCACTCTTCTGCCATTCTA |
| P2-FAM reporter | CCATTATTGCAAATTTC |
| P3-FW | ACATCAAATGTATGCTATTTTCTTGCTTAAAGTG |
| P3-RV | GCTACTTGAGACAAACTGAAAAATTTCTTCT |
| P3-FAM reporter | ACGTGCTCCACTTTGG |
| P4-FW | GTTTTGTCTTTTTCAGAATGCATTTGGTT |
| P4-RV | GGATGCCAGCTATCAACACTGA |
| P4-FAM reporter | CCATTATTGCAAATTTC |
| Rad52-ctr-FW | CCTCATTTTCGTGTGGTTTTGTTGA |
| Rad52-ctr-RV | AGGACCAAAGAGGAACTGGACATAT |
| Rad52-ctr-FAM reporter | CTATCAGCCAGAACCC |
| 15-47530479F (probe) | CCATCTGTATGTGTGTGAGTGGGG |
| 15-47531141R (probe) | AGTTCAGTTTGGCTTGTGCTGTG |
| 15-47528519R | TCTGTCTGCACTCATCCCCTTGA |
| 15-47528244R | TGGGTATTTGGGGGAGCCTTCCT |
| 15-47528695R | GCCCAATATGAGCCTATGCAGGTGG |
| 15-47528244F | TGGGTATTTGGGGGAGCCTTCCT |
| 15-52345375F | TGGCCCATCACTTGGGGTGGA |
| 15-52346061F | TGGCTTCCGTTGCCATGCGA |
| 15-52346301F | AGAGACTGCCTTTGCACACGGG |
| 1-196633183R | TGCCCCCGTTGTCTTCTTGTCA |
| 1-196631132R | CATCTTGGAAGCTTGGGGTA |
| 1-196631489R | CCAACAACGTGCTGTGTGGTTTCC |
| 8-86478509R | TGGGACTTGCTGCGATGGCC |
| 8-86477570R | TGCCCCAAAGCCCGCCAAAA |
| 8-86478926R | GGGTGCCCCCTCATGGCAAG |
| 20-51616829R | CTCCCGAGTATCGAGCCATC |
| 20-51615938R | GCTTTGAGGATGAGTTGGTGC |
| 20-51615795R | AGTTGGCCCAGTGTTTGCAG |

Supplemental Table 4. Oligonucleotide sequences.
